# As you weed, so shall you reap: on the origin of algaculture in damselfish

**DOI:** 10.1186/1741-7007-8-81

**Published:** 2010-06-18

**Authors:** Duur K Aanen

**Affiliations:** 1 Laboratory of Genetics, Wageningen University, PO Box 309, 6700 AH Wageningen, The Netherlands

## Abstract

Within their territories, damselfish cultivate particular algae for consumption. A recent study in *BMC Evolutionary Biology *shows extensive variation among and within fish species in the composition of these algal 'gardens', varying from monocultures to cultures of mixed species, and in the mode of cultivation. This fish-algal agriculture may provide insight into the early stages of domestication.

See research article http://www.biomedcentral.com/1471-2148/10/185

## Commentary

The transition from a hunter-gatherer to an agricultural lifestyle has had dramatic consequences for human evolution [1]. It paved the way for the advanced division of labor, which characterizes modern societies and which was a precondition for their rise. With that, agriculture ultimately is at the basis of the exponential increase in human population size during the last millennium. What is usually less well appreciated is that this transition has meant an exponential increase in the population size of domesticated organisms compared to their free-living ancestors. For example, in the Netherlands there is about one domesticated pig for each human, so that the domesticated animal outnumbers its wild cousin by some three orders of magnitude. Agriculture therefore fully satisfies the criterion for a mutualism: a mutually beneficial interaction between different species.

More traditional forms of agriculture may shed light on the first steps to agriculture. Amerindian farmers propagate cassava (*Manihot esculenta*) clonally by cuttings. However, recombined wild seedlings also appear spontaneously in fields, and farmers allow them to grow. Later, they recruit some large ones for new cuttings, and it has been demonstrated that the selected plants are more heterozygous. Therefore, the farmers (unconsciously) select for increased heterozygosity [2]. This shows that domestication is an ongoing process and that domesticated organisms are not immediately reproductively isolated from their wild relatives. This has also been shown for domestic cattle where introgression of wild Y chromosomes -but not of mitochondria - has occurred in northern Europe, suggesting continued mating with wild bulls after domestication [3]. And on the human side, although a transition back to a hunter-gatherer lifestyle from an advanced agricultural society now seems hard to imagine, there is evidence for a reversal from a primitive agricultural to a hunter-gatherer lifestyle [4].

**Figure 1:**
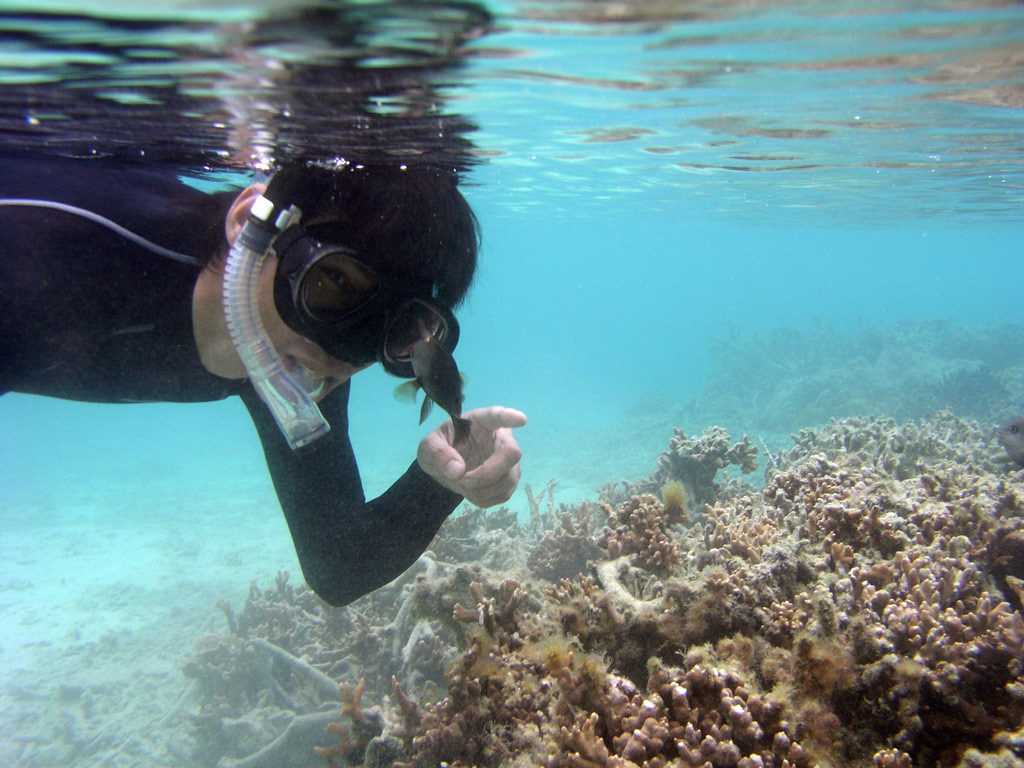
**The researcher and the fish. *Stegastes nigricans *chasing Hiroki Hata off its territory in Okinawa, Japan**. Photograph kindly provided by Hiroki Hata.

A different approach to studying the first stages of agriculture is to look at non-human agricultural mutualisms. Well-known examples are found in two groups of social insects - termites and ants - which have independently domesticated fungi for food [5]. A recent paper by Hata *et al*. [6] in *BMC Evolutionary Biology *describes details of another fascinating agricultural mutualism, this time below the sea surface, between damselfish and filamentous algae. On coral reefs, damselfish individually defend territories from invading grazers and maintain algal turfs, from which they obtain all their food (Figure 1). These turfs are a rich food source and highly productive. Most species grow their algal crops as mixed cultures of mostly Rhodophyta (red algae). The damselfish lack effective enzyme systems or masticatory organs to break down cell-wall material, and hence depend on easily digestible algae. The algae they use typically are fast growers found in the early stages of a succession.

**Figure 2:**
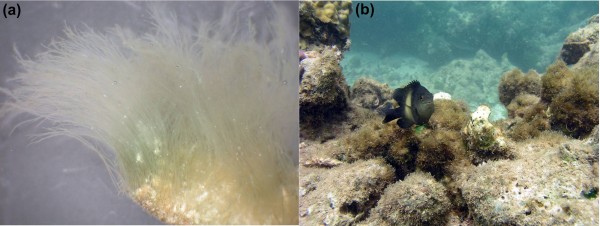
**Variations in algaculture by damselfish**. **(a) **A near monoculture of *Polysiphonia *sp. 1 from a territory of *S. nigricans *in Okinawa. **(b) ***S. nigricans *and its more diverse garden off the coast of Kenya. Photographs kindly provided by Hiroki Hata.

Earlier work by the same group on the damselfish species *Stegastes nigricans *off the coast of Okinawa prefecture in Japan found that it specialized on the filamentous red alga *Polysiphonia *sp. 1, which it cultivated in a near monoculture (Figure 2a). The fish weed out most of the other, indigestible, algae, chase away other herbivores and harvest the alga as their staple food. As this species of *Polysiphonia *is highly susceptible to competition from other algal species, its habitat is limited to *S. nigricans *territories. In this way, the fish and the alga have become highly interdependent and approach the state of an obligate mutualism.

## Algal analysis

Hata *et al*. [6] have now carried out a comprehensive DNA-based analysis of the algal composition of 320 territories from 18 species of damselfish, collected throughout the Indian Ocean and the West Pacific. The authors sketch a more complex picture of the mutualism between *S. nigricans *and *Polysiphonia *sp. 1 than initially believed. While *S. nigricans *populations in Japan do indeed maintain an almost exclusive monoculture of this species, in other parts of the fish's distribution most territories contain more diverse mixed cultures (Figure 2b). This diversity is manifest in two ways. First, in other areas *Polysiphonia *species constitute much less of the total algal biomass per territory; second, in most territories, more than one *Polysiphonia *species is found. Consistent with earlier studies, *S. nigricans *still maintains the most specific association of all damselfish studied: in all 117 territories of *S. nigricans *studied, close relatives of *Polysiphonia *sp. 1 belonging to a single clade were found. Almost all the algae from this clade were exclusively found in territories of *S. nigricans *and never outside damselfish territories, suggesting that this mutualism is obligate for both partners. The 17 other damselfish species studied generally cultivate mixed crops of algae, do this in a more extensive fashion than *S. nigricans*, and have less specific associations with *Polysiphonia *algae.

The monoculture found in the earlier studies was initially interpreted as support for a more derived state of farming in *S. nigricans *than in other damselfish. Selective weeding is unique for *S. nigricans*: other species also chase away competing grazers but they do not weed out unpalatable algae [7]. However, the new study shows that this intensive agriculture does not lead to a monoculture throughout the distribution area of this fish. Variation in the intensity of weeding among regions may explain part of the variation, but the composition of the local algal community might also influence algal composition inside the fish territory.

## Monoculture versus mixed crop

Theoretical studies have indicated that symbiont relatedness may be an important stabilizing factor for mutualisms (for example, see [8]). The basic idea is as follows. A symbiont's fitness can be partitioned into two components: competitive success relative to other symbionts associated with the host, and overall success of the group of symbionts. As relatedness declines within hosts, a symbiont's success depends more on its competitive ability and relatively less on group success. In many cases, competitive traits will have negative side effects for the host, as symbionts will be selected for fast exploitation of the resources provided by the host. Therefore, theory suggests that by selecting for competitive traits symbiont mixing destabilizes mutualisms.

However, we have to consider the biological idiosyncrasies of a particular mutualism to fully appreciate the significance of symbiont relatedness. In the damselfish-alga mutualism, the algae preferred are those that are fast growing and do not invest in persistent cell walls. In the non-mutualistic situation, these algae are found in the early stages of succession. By their continual weeding, *S. nigricans *continuously recreates these early-succession conditions. The main determinant of competitive ability in these early-succession algae is fast growth. And the fast-growing algae are the ones preferred by the fish. Therefore, this form of competition increases rather than decreases the benefit for the host.

## The origin of agriculture

For both humans and non-humans, the transition to agriculture was not a conscious decision. Therefore, we have to identify the consequences of changes occurring in the coevolving organisms that lead to their domestication [1]. Some plausible scenarios have been sketched for domestications by humans. For example, the ears of domesticated wheat and barley do not shatter and drop their seeds when ripe, whereas those of their wild ancestors do, and the seeds are wind dispersed. The non-shattering trait is caused by a single recessive mutation. It seems plausible that humans who collected seeds selected the convenient mutant that concentrated the seeds, and thus selected (unconsciously) for the non-shattering mutation. If some of these seeds were spilled close to human settlements, these mutants gained a fitness benefit as their seeds could be more effectively dispersed, but this depended on intervention by humans [1].

In general, the challenge is to understand the counterintuitive combination of being consumed and simultaneously gaining fitness. A plausible scenario for the origin of termite fungiculture is the initial indiscriminate consumption of unspecialized fungi that inhabited their nest structure. In all extant fungus-cultivating termites, the fungus is propagated by the consumption of nodules, which contain gut-resistant asexual spores, which are mixed with collected plant substrate [9]. It seems plausible that the formation of asexual spores that could survive gut passage was a key innovation in the fungus in response to insect consumption. This change turned the disadvantage for the fungus of being consumed into a benefit [9]. For other insect-fungus mutualisms it has been argued that the interaction started with insects acting as a vector for fungal dispersal [5].

The ancestors of algacultural fish were omnivorous and multiple independent transitions to algaculture have occurred (H Hata, personal communication). This is consistent with the rather unspecialized nature of this domestication. The fish do not plant or inoculate the algae, as has been found in older mutualisms between fungi and insects [5]. Instead, they remove most of the algae from their territories, thereby continuously recreating the early stages of algal succession. The algae thus selected are the algae characterized by fast growth and high palatability. It seems plausible that this mutualism started by weeding or consumption of all the algae in a territory, and that the fast-growing algae remained, and started to specialize on this new niche. With their behavior, the fish show some parallels with cattle, which by grazing create and improve their own niche - grassland. The difference is that the fish seem to remove unpalatable algae without consuming these, and that they are territorial.

## Changes in the 'farmer'

According to the Wikipedia definition of domestication ('the process whereby a population of animals or plants, through a process of selection, becomes accustomed to human provision and control'), the farmer is the active partner and the crop or livestock the passive. However, it is increasingly becoming clear that humans themselves have also undergone extensive genetic adaptations after the adoption of an agricultural lifestyle. For example, the persistence of the ability to digest lactose after the weaning phase has arisen independently multiple times in human populations characterized by a history of cattle herding. Likewise, fungus-growing ants have changed from carnivorous to vegetarian after the adaption of fungiculture. To fulfill the definition of coevolution it remains to be shown in which ways the damselfish have genetically changed in response to changes in their cultivated algae. The recent finding that the gut microbiota of Japanese people contains bacteria that aid in digesting the seaweed present in sushi [10] nicely illustrates the fact that adaptations can occur upon the adoption of seaweed in a diet.

## References

[B1] Diamond J Evolution, consequences and future of plant and animal domestication Nature 2002 418 700 707 10.1038/nature01019 12167878

[B2] Pujol B David P McKey D Microevolution in agricultural environments: how a traditional Amerindian farming practice favours heterozygosity in cassava (*Manihot esculenta *Crantz, Euphorbiaceae) Ecol Lett 2005 8 138 147 10.1111/j.1461-0248.2004.00708.x

[B3] Gotherstrom A Anderung C Hellborg L Elburg R Smith C Bradley DG Ellegren H Cattle domestication in the Near East was followed by hybridization with aurochs bulls in Europe Proc Biol Sci 2005 272 2345 2350 16243693 10.1098/rspb.2005.3243 PMC1559968

[B4] Oota H Pakendorf B Weiss G von Haeseler A Pookajorn S Settheetham-Ishida W Tiwawech D Ishida T Stoneking M Recent origin and cultural reversion of a hunter-gatherer group PLoS Biol 2005 3 e71 15736978 10.1371/journal.pbio.0030071 PMC1044832

[B5] Mueller UG Gerardo NM Aanen DK Six DL Schultz TR The evolution of agriculture in insects Annu Rev Ecol Evol Syst 2005 36 563 595 10.1146/annurev.ecolsys.36.102003.152626

[B6] Hata K Watanabe K Kato M Geographic variation in the damselfish-red alga cultivation mutualism in the Indo-West Pacific BMC Evol Biol 2010 10 185 10.1186/1471-2148-10-185 PMC2905425 20565824

[B7] Hata H Kato M Monoculture and mixed-species algal farms on a coral reef are maintained through intensive and extensive management by damselfishes J Exp Mar Biol Ecol 2004 313 285 296 10.1016/j.jembe.2004.08.009

[B8] Frank SA Host-symbiont conflict over the mixing of symbiotic lineages Proc Biol Sci 1996 263 339 344 10.1098/rspb.1996.0052 8920255

[B9] Aanen DK As you reap, so shall you sow: coupling of inoculating and harvesting stabilizes the mutualism between termites and fungi Biol Lett 2006 2 209 212 17148364 10.1098/rsbl.2005.0424 PMC1618886

[B10] Hehemann JH Correc G Barbeyron T Helbert W Czjzek M Michel G Transfer of carbohydrate-active enzymes from marine bacteria to Japanese gut microbiota Nature 2010 464 908 912 10.1038/nature08937 20376150

